# Tweeting for Health Using Real-time Mining and Artificial Intelligence–Based Analytics: Design and Development of a Big Data Ecosystem for Detecting and Analyzing Misinformation on Twitter

**DOI:** 10.2196/44356

**Published:** 2023-06-09

**Authors:** Plinio Pelegrini Morita, Irfhana Zakir Hussain, Jasleen Kaur, Matheus Lotto, Zahid Ahmad Butt

**Affiliations:** 1 School of Public Health Sciences Faculty of Health University of Waterloo Waterloo, ON Canada; 2 Department of Systems Design Engineering University of Waterloo Waterloo, ON Canada; 3 Research Institute for Aging University of Waterloo Waterloo, ON Canada; 4 Institute of Health Policy, Management, and Evaluation University of Toronto Toronto, ON Canada; 5 Centre for Digital Therapeutics Techna Institute University Health Network Toronto, ON Canada; 6 Department of Data Science and Business Systems, School of Computing College of Engineering and Technology SRM Institute of Science and Technology Kattankulathur India; 7 Department of Pediatric Dentistry, Orthodontics and Public Health Bauru School of Dentistry University of São Paulo, Bauru Brazil

**Keywords:** big data, deep learning, infodemics, misinformation, social media, infoveillance

## Abstract

**Background:**

Digital misinformation, primarily on social media, has led to harmful and costly beliefs in the general population. Notably, these beliefs have resulted in public health crises to the detriment of governments worldwide and their citizens. However, public health officials need access to a comprehensive system capable of mining and analyzing large volumes of social media data in real time.

**Objective:**

This study aimed to design and develop a big data pipeline and ecosystem (UbiLab Misinformation Analysis System [U-MAS]) to identify and analyze false or misleading information disseminated via social media on a certain topic or set of related topics.

**Methods:**

U-MAS is a platform-independent ecosystem developed in Python that leverages the Twitter V2 application programming interface and the Elastic Stack. The U-MAS expert system has 5 major components: data extraction framework, latent Dirichlet allocation (LDA) topic model, sentiment analyzer, misinformation classification model, and Elastic Cloud deployment (indexing of data and visualizations). The data extraction framework queries the data through the Twitter V2 application programming interface, with queries identified by public health experts. The LDA topic model, sentiment analyzer, and misinformation classification model are independently trained using a small, expert-validated subset of the extracted data. These models are then incorporated into U-MAS to analyze and classify the remaining data. Finally, the analyzed data are loaded into an index in the Elastic Cloud deployment and can then be presented on dashboards with advanced visualizations and analytics pertinent to infodemiology and infoveillance analysis.

**Results:**

U-MAS performed efficiently and accurately. Independent investigators have successfully used the system to extract significant insights into a fluoride-related health misinformation use case (2016 to 2021). The system is currently used for a vaccine hesitancy use case (2007 to 2022) and a heat wave–related illnesses use case (2011 to 2022). Each component in the system for the fluoride misinformation use case performed as expected. The data extraction framework handles large amounts of data within short periods. The LDA topic models achieved relatively high coherence values (0.54), and the predicted topics were accurate and befitting to the data. The sentiment analyzer performed at a correlation coefficient of 0.72 but could be improved in further iterations. The misinformation classifier attained a satisfactory correlation coefficient of 0.82 against expert-validated data. Moreover, the output dashboard and analytics hosted on the Elastic Cloud deployment are intuitive for researchers without a technical background and comprehensive in their visualization and analytics capabilities. In fact, the investigators of the fluoride misinformation use case have successfully used the system to extract interesting and important insights into public health, which have been published separately.

**Conclusions:**

The novel U-MAS pipeline has the potential to detect and analyze misleading information related to a particular topic or set of related topics.

## Introduction

### Background

Big data refers to large volumes of internet data produced and consumed by people with varying levels of complexity and ambiguity and generated at varying velocities, which includes health data [1]. In this scenario, Eysenbach [2] proposed the concept of infodemiology, described as “the science of distribution and determinants of information in an electronic medium and its effects on individual and public health.” Specifically, the big data originating from users’ health information–seeking behavior on search queries and social media may inform the planning and implementation of public health measures [3,4]. Interestingly, social media data are constantly increasing, which supports the users’ interest in expressing their concerns, doubts, and advice about health conditions [4,5]. Moreover, the assessment of real-time health content has substantially contributed to the surveillance and forecasting of diseases, outbreaks, and epidemics [6]. Previous studies [4,7-11] have demonstrated that the primary infodemiological social media data sources arise from Twitter, Facebook, and Instagram despite established limitations such as user demographics, time-specific data extraction, and data samples representative of the entire user population [12-14]. Nevertheless, these data can rarely be processed and analyzed using traditional methods, algorithms, or commercial frameworks, which often results in a gap between the epidemiological capacity of these data and the public health officials who do not have immediate access to these data [15].

Concurrently, the overabundance of digital health content makes it difficult for the public to distinguish trustworthy from false or misleading information [16]. It is noteworthy that most users consume and share social media content without checking its trustworthiness in depth [17,18]. Furthermore, people exposed to health-related falsehoods have a propensity to develop harmful health beliefs, which negatively influence personal decision-making and public health outcomes [19]. For example, it is estimated that vaccine hesitancy that originated from immunization-related misinformation caused a loss of at least US $50 million each day in the United States since the vaccines became widely available, in addition to the loss of thousands of lives [20].

Information disorders have a range of definitions in the literature, which include misinformation, malinformation, disinformation, fake news, and conspiracy theories [21]. For this paper, the authors used “misinformation” as an umbrella term, covering any false or misleading information regardless of the source or intent [18,19]. In this way, the authors proposed a feasible classification model as the distinction of types of information disorder requires the definition of distinct subjective information aspects, potentially decreasing accuracy and increasing false positives [21,22].

### Objective

It is necessary to develop an expert system for researchers, public health officials, and related policy makers that can handle large amounts of data related to health information and can identify and predict trends in public health misinformation, allowing for government intervention before public health crises emerge [23,24]. To address this need, this study aimed to design and develop a big data pipeline and ecosystem named UbiLab Misinformation Analysis System (U-MAS) to identify and analyze falsehood regarding distinct health issues on social media. Although this architecture was developed to detect and understand misinformation trends for public health outcomes, it can be used for misinformation related to any topic if a small subset of expert-validated data exists.

## Methods

### Technology Framework

U-MAS is a platform-agnostic ecosystem built primarily in Python and relies on the Twitter V2 application programming interface (API) [25] as well as the Elastic Stack [26], specifically Elasticsearch and Kibana. Moreover, U-MAS has been designed and developed to run on a virtual machine (VM) with modest memory and computing requirements. The user can choose the keywords, search time frame, and data properties of tweets to be extracted into JSON [27] files using the developed big data U-MAS. In addition, the raw JSON files are stored in a repository in Azure Binary Large Objects (BLOB) storage [28]. Finally, the Python libraries *Pandas* [29] and *NumPy* [30] have been used to clean the system and preprocess the data for better accuracy in the analysis stage.

### Data Set Used

This study relied on 2 sets of data, one from Instagram and the other from Twitter. During the conception of U-MAS, the previously expert-validated Instagram data set (N=500) collected using CrowdTangle was used to design and validate the system architecture [4]. Initially, the system architecture involved uploading a CSV or JSON data file for further analysis. As each system component was developed, the study moved to direct querying from the Twitter V2 API [25]. Both data sets were related to fluoride misinformation and had 500 instances each. However, because the Twitter data set is under qualitative review by the investigators, some system components are still previously developed components using the Instagram data set. We do not anticipate this to have an impact on the system’s accuracy because the search strategies of both data sets were designed to ensure that the data and components developed from the data could be compared and integrated with one another accurately despite having different userbases.

### System Design and Architecture

#### Overview

**Figure 1:**
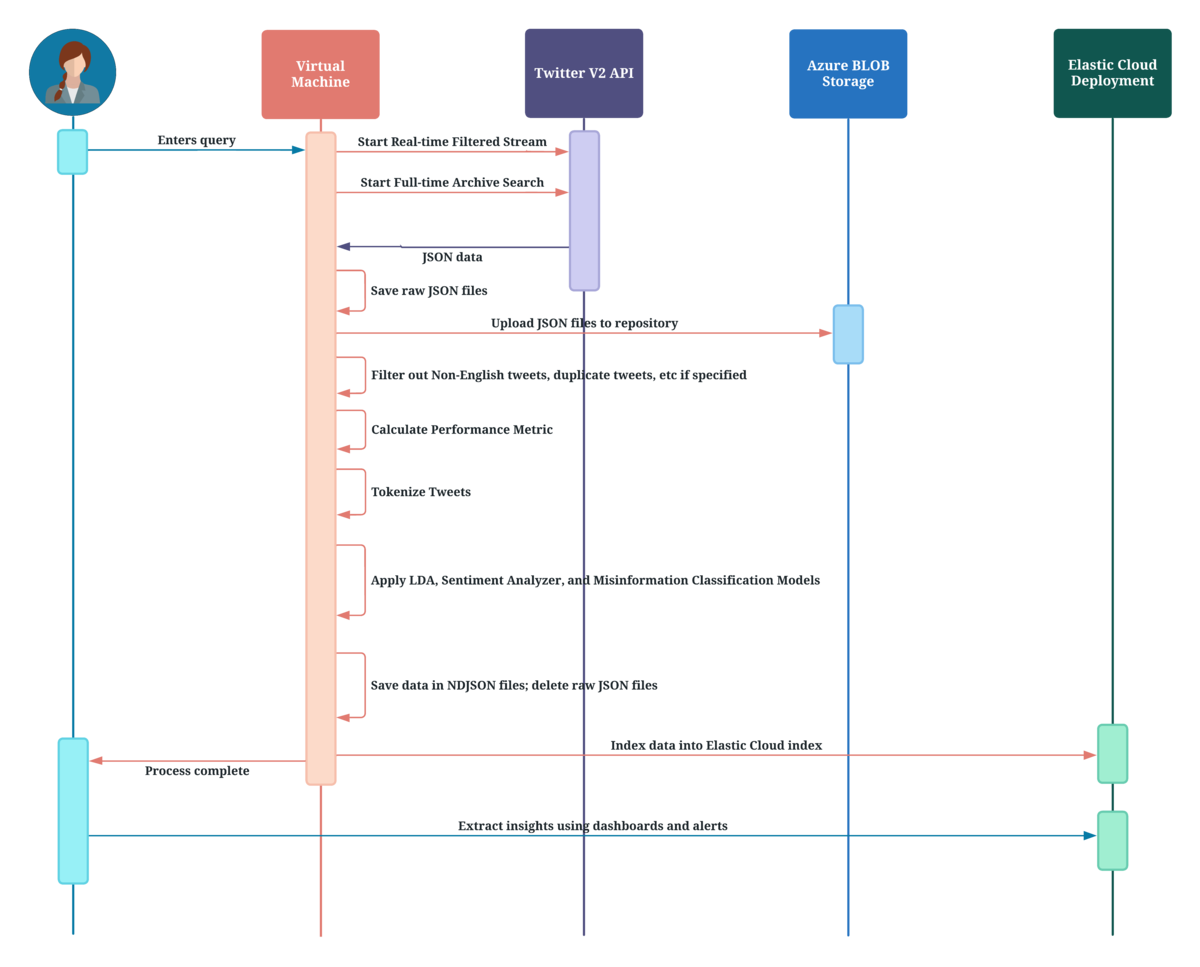
Sequence diagram representing the working of UbiLab Misinformation Analysis System (U-MAS). API: application programming interface; BLOB: Binary Large Objects; LDA: latent Dirichlet allocation.

This infodemiological and methodological study describes the design and development of a big data pipeline and ecosystem called U-MAS to identify and analyze false or misleading health information on social media. The U-MAS pipeline comprises five components: (1) data extraction framework, (2) latent Dirichlet allocation (LDA) topic model, (3) sentiment analyzer, (4) misinformation classification model, and (5) Elastic Cloud deployment (indexing of data and visualizations). Initially, the expert-validated data are used to train and load an LDA model, a sentiment analyzer, and a misinformation detection model into the U-MAS pipeline. The defined models are applied to the preprocessed data, and the results are added to the data saved in the Newline Delimited JSON (NDJSON) files. Finally, the NDJSON files are parsed and loaded into an index on the Elastic Cloud deployment component. Hence, the index data can be displayed in dashboards with advanced visuals and analytics relevant to the misinformation analysis. Figure 1 illustrates how the ecosystem will be provided with data from Twitter about a specific overarching topic using a data extraction framework.

It is noteworthy that the Instagram data informed the initial training of the models [4]. Although the LDA topic model trained from the Twitter data (N=500) related to fluoride misinformation had been validated, the sentiment analyzer and misinformation classifier were awaiting manual qualitative analysis to be validated against. Thus, this study used the previously validated Instagram models for sentiment analysis and misinformation classification.

#### Data Extraction Framework

**Figure 2:**
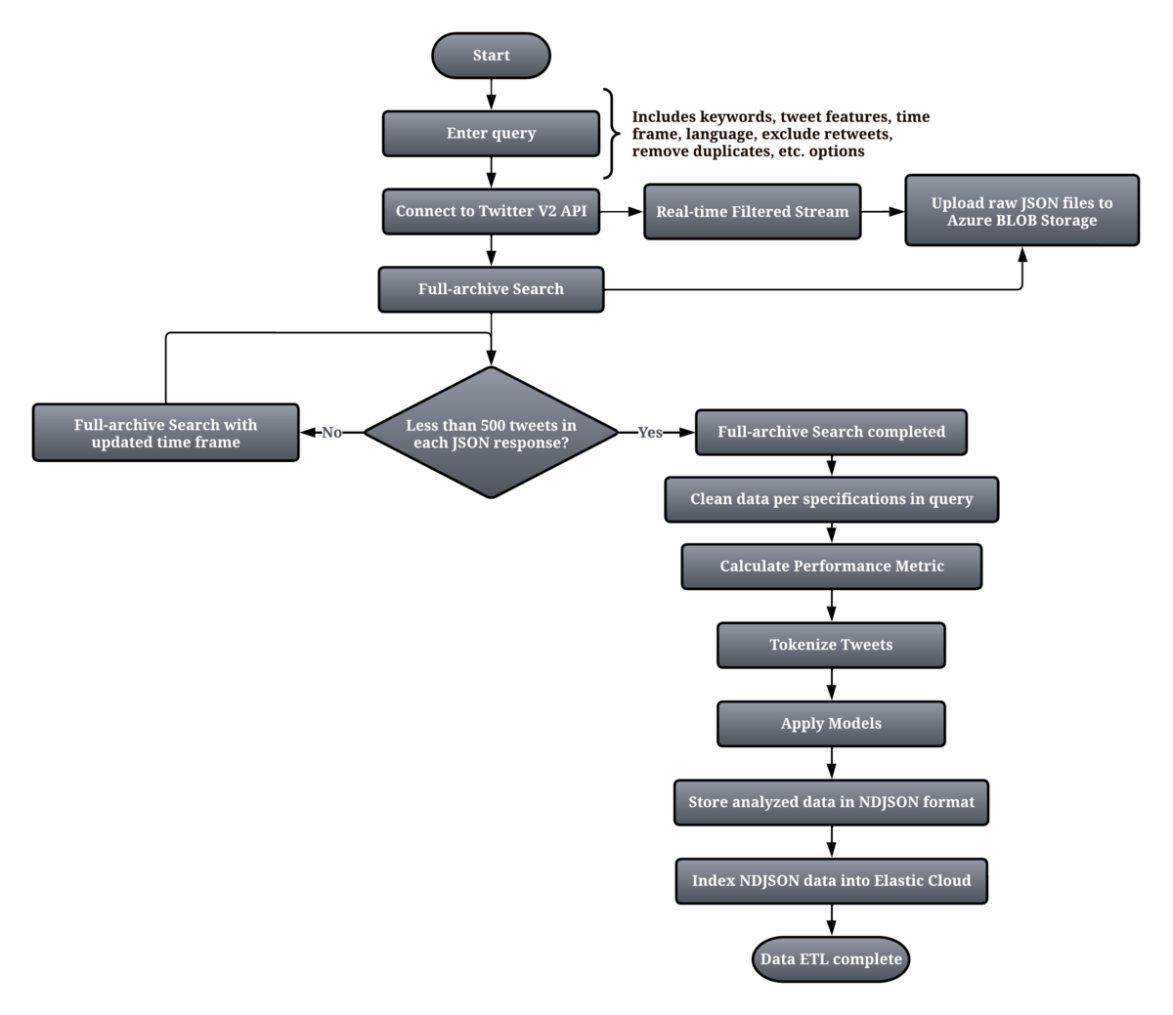
Working flow diagram of the data extraction framework. API: application programming interface; BLOB: Binary Large Objects; ETL: Extract, Tranform, Load; NDJSON: Newline Delimited JSON.

The dependencies of the Python-based data extraction script primarily consist of two files: (1) *twitter-keys.txt* (which holds the bearer token for authorization) and (2) *twitter_meta.txt* (which contains information on each of the 1-month time frames that the API will query for improved file storage and computing power). Figure 2 depicts the complete process performed by the Python-based data extraction script as it runs on the VM.

Academic researchers are given special access to the Twitter V2 API, which provides accounts with a researcher level of access to Twitter’s real-time and historical public data, as well as additional features and functionality that allow for the collection of more precise, complete, and unbiased data sets. Furthermore, the researcher level of access allows for (1) a monthly tweet cap of 10 million tweets; (2) up to 1000 streaming query rules of up to 1024 characters; (3) streaming rates of 50 requests per 15 minutes per app; and (4) entire range of search operators, allowing for searches of up to 1024 characters [25].

A basic framework has been created to retrieve tweets from the full-archive search end point. On the basis of the search query, the end point enables researchers to programmatically access public tweets from the entire archive dating back to the first tweet in March 2006. The end point can send up to 500 tweets in reverse chronological order per request, with pagination tokens available for paging through huge collections of matching tweets. At the same time as the full-archive search, if the user specifies the option, a real-time stream is also set up using the filtered stream end point. The raw results are saved in JSON files and uploaded into a designated storage container in Azure BLOB storage.

Following the subsequent storage in the repository, the raw data are further preprocessed in accordance with the user’s requirements. The user may specify the exclusion of non-English language tweets, retweets, or duplicate tweets (which have the same content but may be authored at different times or by different people). Moreover, the user may also specify the inclusion of tweets from geolocations such as Toronto, Ontario; Vancouver, British Columbia; or Canada as a whole. A basic performance metric that assigns weights to each public metric, such as like count, reply count, quote count, and retweet count, can be computed at this stage. This allows the filtering of duplicate tweets by retaining the one with the highest performance score associated with it.

After the validation by a subject matter expert, a data set including N instances of the highest-performing tweets was created to inform the training of the LDA and misinformation classification models. Further preprocessing of the words was performed to assure the quality of the topic modeling analysis, including the removal of symbols, special characters, punctuation, URLs, digits, personal pronouns, other stop words, query keywords, and platform-specific language.

#### Topic Modeling (LDA Analysis)

Topic modeling is an unsupervised learning algorithm that is used in the realm of machine learning to extract groupings of keywords from a large corpus of unstructured and unlabeled documents, including health information [31,32]. These groupings can be loosely termed as topics, where each topic is a probability distribution of how often particular keywords may appear together in a document that falls under that topic. The LDA is a topic modeling algorithm that can be used to determine the salient terms of a corpus as well as the topics within that corpus [31]. Given the number of topics *K*, the LDA algorithm will generate a probability distribution of the relevancy of the words attributed to the topic for each topic. The U-MAS pipeline used the framework for LDA analysis developed for Twitter data to train *K* LDA models for *K* topics by using a sample of the 500 highest-performing tweets. This framework first determines the best range of *K* values within a larger range of *K* values, from *K*=2 to *K*=50, using 5-fold cross-validation, a process in which the training data are split into 5 subsets, and the model is trained on all but one of these subsets; the remaining subset is used to validate the model [31]. After identifying a smaller range of possibly optimal *K* values, the topic models for each of these *K* values and their respective coherence scores are computed. The Python-based *Gensim* [33] and *spaCy* [34] libraries have been used for topic modeling and advanced natural language processing (NLP). In addition, an expert on the topic of discussion validates the topic model with the highest coherence value by rating the model on the meaningfulness of its topics, how well publications within a single topic are related, and how publications of different topics are distinct from each other [35,36]. The remaining data set is automatically categorized using the model once a satisfactory topic model has been created. Each tweet is assigned a topic classification and the likelihood that the tweet will fall under that category.

#### Sentiment Analysis

##### Overview

Sentiment analysis is a category of NLP tasks under opinion mining [37]. It entails classifying the text as positive, neutral, or negative and can be dichotomized into positive and negative for class imbalance considerations [38]. For simple sentences such as, “This class is great!” (positive) and “I wish this class would just get over already” (negative), the average sentiment analyzer should be able to correctly classify the emotion behind these texts. There are a few major hurdles to sentiment analysis in the context of social media. First, most sentiment analyzers struggle with sarcasm (“Wow! You’re so smart”). Second, sentiment analyzers may be unable to determine whether the people or things mentioned in a text are liked or disliked. Finally, in the setting of social media, people are more inclined to express strong views than neutral ones and may be overwhelmingly more positive than negative, depending on the platform [39,40]. In the initial stages of the development of the U-MAS pipeline, 2 of the current main possibilities of NLP-grounded sentiment analysis have been applied, that is, the Valence Aware Dictionary and Sentiment Reasoner (VADER) and the Bidirectional Encoder Representations from Transformers (BERT). One study [41] showed that other commonly used sentiment analysis methods, such as lexicon-based approaches (eg, Sentiwordnet) or machine learning approaches (eg, TextBlob), fail to perform well on the slang, sarcasm, and negation that frequently appears on social media. The first approach represents the classifier commonly used in web-based health information sentiment analysis [39,40,42], whereas the second approach has become more successful and has been adopted in more recent literature [43-45].

##### VADER Algorithm

The VADER algorithm is a lexicon- and rule-based sentiment analysis tool that is specifically attuned to sentiments expressed in social media [46]. It responds to the polarity and strength of sentiment in a publication, handling conventional internet language, emoji, and the use of punctuations and capitalization for sentiment modification. Although VADER presented a limited accuracy compared with deep learning sentiment analyzers, its interpretability is friendly to nonspecialists [47]. In contrast, when we performed an exploratory analysis using VADER on the studied data set [4], we found that despite classifying positive sentiment well, the algorithm fell short in recognizing negative or neutral sentiments. In fact, the Matthews correlation coefficient (MCC) [48,49] was generated (which was approximately 0.327) to assess the algorithm’s accuracy owing to the data set’s extreme class imbalance. Moreover, posts that were categorized as political misinformation and nonpolitical misinformation had their MCC calculated. The correlation coefficient for nonpolitical misinformation posts was only 0.149. The correlation coefficient for political misinformation posts was comparable with its VADER counterpart (0.327). However, it is important to note that from the qualitative analysis, it was found that nonpolitical misinformation posts were largely positive in nature, whereas political misinformation posts were often negative or neutral [4].

##### BERT Algorithm

**Figure 3:**
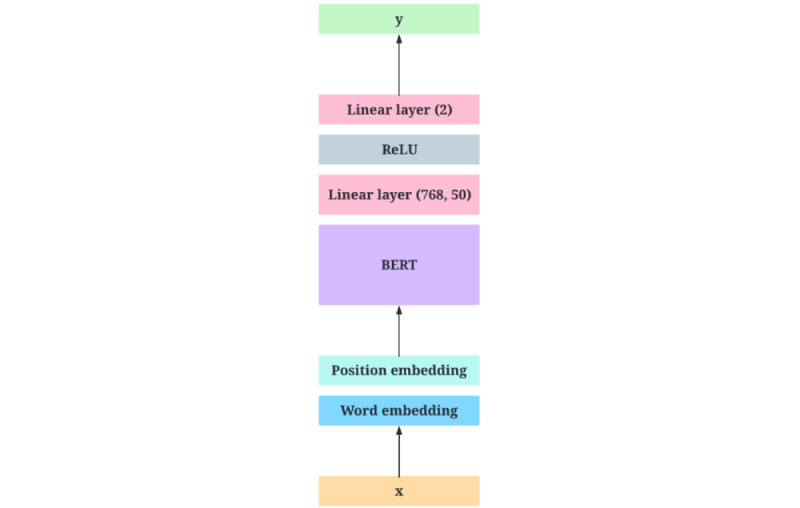
The architectural design of sentiment analysis model, where x is the text and y is the sentiment. BERT: Bidirectional Encoder Representations from Transformers; ReLU: Rectified Linear Unit.

Another approach was using a small subset of independently validated data to create a BERT model specifically for the data set’s sentiment analysis. The BERT model can handle a wide range of NLP tasks, including language inference, question answering, and text detection [50,51]. As a result of its training process, the BERT model develops an understanding of word contextual embeddings (word embeddings and position embeddings). In other words, BERT [50] learns the connotation of each word based on its position in the sentence. The BERT model can be modified to learn specific tasks using transfer learning, with only minor changes to the model’s actual architecture. In this situation, a simple BERTBASE [50,51] (uncased) model was trained with some fine-tuning of the last layers for the sentiment analysis task. At the BERTBASE (uncased) model output, a simple rectified linear unit (ReLu) activation function [52] classifier was applied to produce either positive (0) or negative results (1). ReLu [52] is a simple piecewise linear function that returns 0 for all negative values *v* and is equal to *p × v* for positive values of *v*, where *v* is the value at each neuron and *p* is the learnable parameter (in this case, whether the text is negative or not). Therefore, ReLu is more computationally efficient than other popular activation functions and mitigates the vanishing gradient problem. The architectural design for this model is illustrated in Figure 3.

Because most social media data are positive in nature, the model has been trained to be more sensitive to negative or neutral sentiments. Table 1 displays the initial results of the 2 sentiment analyzers on the data set. The BERT sentiment analyzer clearly outperformed the VADER model in the model’s initial run. It initially had an MCC of 0.478, with equally high coefficients for the dichotomized groups of misinformation posts (0.33) and political misinformation (conspiracy theory) posts (0.35). After further training, the BERT sentiment analyzer model’s overall correlation coefficient value increased to 0.72. The sentiment analyzer model was applied to the entire data set in the U-MAS pipeline.

**Table 1:** Comparison of the initial performance of Valence Aware Dictionary and Sentiment Reasoner (VADER) and Bidirectional Encoder Representations from Transformers (BERT) for sentiment analysis.

Algorithm	Matthews correlation coefficient		
	All	Nonpolitical misinformation	Political misinformation
VADER	0.327	0.149	0.327
BERT	0.478	0.33	0.35

#### Misinformation Classification

The second modeling task involved misinformation classification. As mentioned above, U-MAS can identify real-time health misinformation on social media about a specific topic from an initial expert-based analysis. This evaluation allows for the characterization of these falsehoods grounded on the social media users’ interests. The definition of the users’ interests enables the dichotomization of health misinformation between nonpolitical and political [4]. This differentiation is desirable because spreading falsehoods relates to users’ ideological motivations and political polarization [18,53,54]. Specifically, misinformation is often formulated to discourage the use of public health policies, such as vaccine campaigns and supply water fluoridation [4,19,55]. In this sense, the distinction of political misinformation can provide a selection of falsities that singularly affect public health outcomes.

**Figure 4:**
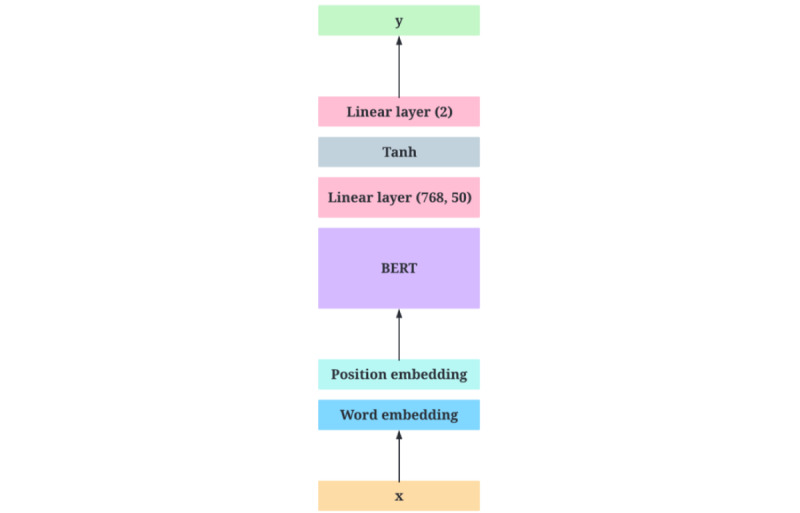
The architectural design of the misinformation classification model, where x is the text and y is the category of misinformation. BERT: Bidirectional Encoder Representations from Transformers.

In the Instagram data set, the dichotomization was also incredibly imbalanced, with 413 posts categorized as misinformation and the remaining 77 posts categorized as political misinformation by independent experts [4]. As a result, the classifier was trained to learn to predict political misinformation (1) over misinformation (0). Again, a simple BERTBASE (uncased) model [50,51] was trained with the last fine-tuned layers. The output of the BERT model was fed into a simple hyperbolic tangent (tanh) classifier, which determines whether the text’s misinformation is due to political disinformation or general misinformation. Tanh activation is preferred to sigmoid solely because it is centered at the origin and thus has a gradient 4 times the sigmoid function’s gradient, mitigating the vanishing gradient problem. Because the data set was class imbalanced, the MCC was again computed between the human classification and the model’s predictions, which produced a result of 0.819 instead of a confusion matrix or *F*_1_-score. U-MAS used this classification model to categorize the data as either political misinformation or not once the sentiments of the texts in the data set had been categorized. The architectural design of this model is shown in Figure 4.

#### Elastic Cloud Deployment

Elastic Cloud is the managed cloud deployment variant of the Elastic Stack, which consists of Elasticsearch and Kibana as its 2 primary system features [26,56,57]. Elasticsearch is a distributed, RESTful search and analytics engine that supports strong analytics and quick accurate search [56]. It can easily store massive amounts of data, including numbers, text, geolocation, and structured and unstructured data. It is ideally suited for analyzing social media data mapped to various data models (ie, from different social media platforms).

Kibana is a graphical user interface–based analytics and management platform that enables the diverse analysis of data stored in Elasticsearch through complex visualizations, dashboards, machine learning tasks, and cloud deployment management. Standard visualizations such as charts, tables, metrics, and gauges are supported by Kibana, along with geospatial analysis using maps, time series analysis, anomaly detection, and graphs or networks [57]. Kibana also supports Vega Lite, a scripting language that can design custom visualizations including a word cloud.

The analyzed data are stored in NDJSON files after the model analyses are completed, and each NDJSON file is parsed using the Elastic Indexing Framework. The developed Python script depends on *elastic-cloud.txt*, which contains the Cloud ID and password required to access the Elastic deployment from the VM. The VM connects to the deployment after reading the data from the *elastic-cloud.txt* file. The process then loops through the NDJSON files, indexing each JSON file into the appropriate Elastic index. Once the data have been properly indexed into the Elastic Cloud deployment, a secure, real-time, interactive analytics front end of the system can be built using Kibana’s dashboard functionalities. The Kibana dashboard can be designed such that both novice and experienced users can readily understand the visualizations and metrics. For the specific purpose of misinformation analysis, the dashboard can include time series data visualizations and analysis, topic distributions and explanations from the results of the LDA analysis, and pie chart visualizations of the distribution of sentiment analysis results and misinformation classification models. Moreover, the dashboards (Multimedia Appendix 1) can illustrate correlations between any of these variables and a word cloud of salient terms in the data.

#### Expert Validation

There are 4 phases of expert validation used in this study. First, the subject matter experts performed a manual qualitative analysis on a subset of N instances from the data set. For the fluoride misinformation use case, this sample of data was the 500 highest-performing posts collected from Instagram and 500 highest-performing tweets collected from Twitter [4]. We define performance based on the total number of interactions on the publication, that is, the performance metric. The qualitative analysis for the fluoride misinformation use case performed by trained subject matter experts included identifying the type of misinformation (political or nonpolitical) present in the publication as well as determining the sentiment (positive, negative, or neutral) of the publication [4]. These results acted as the training labels for the misinformation classification model and sentiment analyzer. The next phase involved validating the topics identified by the LDA topic models by determining whether the topics were semantically meaningful and the overarching themes of each topic from the publications categorized within them. Details on the expert validation performed on the Instagram data set of the fluoride misinformation use case are further elaborated in a separate publication [4]. The third phase concerned the overall testing of the system for ease of use and automated analysis capabilities. This included the design of visualizations for dashboards, metrics displayed as part of the analytics, and ease of navigation on the Elastic Cloud deployment. Finally, the experts would periodically evaluate a sample of new publications collected through the system to ensure the validity of the automated analysis of the system.

### Evaluation Metrics

The coherence score and MCC are the evaluation metrics used to determine the performance of the U-MAS components.

#### Coherence Score

Topic coherence is a measure of how semantically similar high-scoring words within a single topic are to each other [36]. A high coherence score is generally a good indication of human-interpretable topics compared with other performance metrics associated with topic modeling. One popular metric is perplexity [58], which can be summarized as a measure of how well a topic model would perform on new data it has not seen before. Although perplexity has been commonly used in the literature, recent studies have shown that perplexity and human judgment are poorly correlated and can sometimes be anticorrelated [59].

#### MCC Evaluation

Deep learning models in the literature are usually assessed using common performance metrics such as accuracy and *F*_1_-score [60]. Unfortunately, both measures may be hyperinflated indicators of performance when the data set on which the model is trained on is severely class imbalanced [61], such as the data set of the fluoride misinformation use case [4]. In the case of class-imbalanced data sets, MCC, Cohen κ score [62], and Brier Loss score [63] are the most frequently used performance metrics. However, recent studies comparing the efficacy of these 3 metrics across important border cases have indicated that the MCC should be the preferred evaluation metric for binary classification problems on imbalanced data sets (Table 2) [64].

**Table 2:** Summary of the evaluation metrics and their respective interpretation.

Evaluation metric	Range of values	Definition	Interpretation
C_v^a^	0 to 1	Measures how semantically similar words within a topic are to each other	A good and generally achievable range is 0.5<C_v<0.8.
Accuracy	0 to 1	Indicates the ratio between the number of correct predictions and the total number of predictions	Accuracy=1 indicates no incorrect predictions.
*F*_1_-score	0 to 1	The harmonic mean of a model’s ability to correctly predict positive instances (recall) and minimize predicting negative instances as positive (precision)	F1-score=1 indicates that the model perfectly predicts positive instances and does not misclassify negative instances as positive.
MCC^b^	−1 to 1	Measures the relationship between the number of positive instances correctly classified, the number of negative instances correctly classified, and the number of positive and negative instances misclassified	MCC=−1 indicates a perfectly inaccurate classifier. MCC=0 indicates a perfectly random classifier. MCC=1 indicates a perfectly accurate classifier.
Cohen κ	0 to 1	Measures the interrater agreement of 2 raters (in machine learning, this is the classifier and the ground truth)	Cohen κ=0 indicates no agreement. Cohen κ=1 indicates perfect agreement.
Brier Loss	0 to 1	A cost function that measures the difference between the predicted probability and the ground truth	Brier Loss=0 indicates perfect accuracy. Brier Loss=1 indicates perfect inaccuracy.

^a^C_v: coherence score.

^b^MCC: Matthews correlation coefficient.

### Data Security and Confidentiality

U-MAS ensures data security through Kibana’s built-in user roles and permissions management system. A limited group of individuals with administrator privileges and read or write access to Elasticsearch (which manages the actual storage of the data) and Kibana (which handles the analytics and visualizations of the data) can configure and administer the system. In addition, the users with administrative privileges may be able to add users to the deployment and define their roles. However, users who will only interact with the dashboard to make informed decisions about the misinformation identified and analyzed can be assigned a role that allows them to only read the data or interact with the visualizations and analytics.

### Ethical Considerations

This study did not require institutional review board approval from the University of Waterloo, Office of Research Ethics because federal regulations do not apply to research using publicly available data that do not involve human participants.

## Results

At its most current iteration, U-MAS is being used to detect and analyze fluoride-related misinformation [4]. In addition, U-MAS is currently being adapted to analyze vaccine- and heat wave–related misinformation, providing real-time insights about their trends. Table 3 summarizes the current iteration of the system components performance with respect to the fluoride misinformation use case, as discussed in the earlier chapters [4]. The data extraction framework for Twitter has been used to collect thousands of documents in a relatively short period (from 30 s to 15 min depending on the number of documents). For the fluoride misinformation use case, the data extraction framework successfully extracted 32,000 documents, spanning a period of 5 years, in approximately 1 minute. The LDA models have attained relatively high coherence scores. The sentiment analyzer and misinformation classifier also performed well but could be improved in future iterations.

**Table 3:** Summary of the system components performance with respect to fluoride misinformation.^a^

Component				Instagram		Twitter
**Data extraction framework**						
	Status		In development		Complete and in use	
	Performance		—^b^		Satisfactory, extracted over 32,000 tweets from a period spanning 5 years in 1 minute	
**LDA^c^ topic model**						
	Status		Complete and validated		Complete and validated	
	**Performance**					
		Coherence score	0.54		0.54	
		Number of topics	7		3	
**Sentiment analysis**						
	Status		Complete		In development	
	**Performance**					
		Matthews correlation coefficient	0.717		—	
		Accuracy	0.92		—	
		*F*_1_-score	0.765		—	
		Brier Loss score	0.08		—	
		Cohen κ score	0.717		—	
**Misinformation classification**						
	Status		Complete		In development	
	**Performance**					
		Matthews correlation coefficient	0.819		—	
		Accuracy	0.95		—	
		*F*_1_-score	0.848		—	
		Brier Loss score	0.05		—	
		Cohen κ score	0.819		—	

^a^Complete system design and pipeline: status—integrated for Twitter data; performance—satisfactory, meaningful insights extracted by independent investigator.

^b^Not available.

^c^LDA: latent Dirichlet allocation.

## Discussion

### Principal Findings and Practical Implications

This paper highlights the design and development of a novel big data pipeline and ecosystem for detecting and analyzing misinformation related to a particular topic or set of related topics. Using a data extraction methodology, the ecosystem ingested information from Twitter regarding a particular overarching topic. In this context, preprocessing techniques and topic modeling (LDA analysis) have been applied to the given data to categorize the topics quickly. Furthermore, the sentiment analysis and misinformation detection BERT models have also been used, and when combined, these models have been used to monitor real-time trends in the dissemination of information about specific topics. In addition, a sample of the data has been periodically validated by independent experts, enabling the retraining of the corresponding models (LDA, sentiment, and misinformation detection) for increased accuracy within the ecosystem. The ecosystem has been implemented for the fluoride misinformation use case, and the details of the insights extracted using the system by the independent expert have been published separately [4].

The novel U-MAS pipeline has the potential to revolutionize the public health space. Researchers, public health officials, and policy makers can use this system to capitalize on the ever-increasing number of people globally using social media to discuss various important health topics. Moreover, this system enables users without a technical background to focus on data analysis concerning public health as opposed to concerning themselves over the technical aspects of obtaining, transforming, and storing the data for analysis. U-MAS has several uses for governments to prevent misinformation in the infodemiology and infoveillance context. At the present condition of the system, U-MAS can easily be implemented to discover the most prevalent talking points of the subset of the population that distrust or purposefully denigrate important public health measures for financial or political interests [4]. Furthermore, U-MAS can track the relevance of these topics over time and provide insight into the campaigns against health misinformation that should be targeted. Finally, U-MAS can determine the efficacy of such campaigns through various time series analysis and metrics on topic and keyword relevance.

The authors anticipate ongoing testing and validation of the system even as it moves into production, as the users of this expert ecosystem will be researchers and government entities who aim to prevent public health disasters by ensuring that the public receives accurate information on the web. Thus, this system is currently being tested internally for several health use cases, including vaccine hesitancy (2007-2022) and heat illness–related misinformation analysis (2011-2022).

### Limitations and Future Work

U-MAS faces several limitations that will be the focus of future iterations of the system. First, the expert validation of the data set is time-consuming, and measures must be taken to ensure that the data sample is representative of the entire media platform’s universe. As periodic validation of data is essential to ensure the high performance of U-MAS, a data-independent sentiment analyzer is currently being developed to reduce the time spent on the manual evaluation process. Moreover, although the system recognizes when the content of publications has URLs to external sources, it currently does not extract information on what these external sources contain. It also uses public interactions as the metric for the relevancy of a misinformation trend, but studies have shown that many social media users are passive and scroll without necessarily publicly interacting with the publication they encounter. Unfortunately, most social media platforms do not publicly show the view count or the reach of a textual publication. Future work could include incorporating view counts from video publications, such as Instagram reels or TikTok. However, studies have also shown a positive correlation between the number of public interactions and total reach of a publication. Hence, the authors believe that the relevancy analysis provided by U-MAS is still accurate despite this limitation.

Furthermore, the system does not account for automated accounts that pose as real human users, or social bots, which are known to proliferate misinformation about controversial political and public health matters. For example, studies [65-67] have shown that during the pandemic, COVID-19 misinformation on Twitter was more likely to come from social bots than from human users and that they often pushed conspiracy theories (political misinformation). It would be prudent in the next iteration of the system to include bot detection as a component in the U-MAS system to identify prominent health misinformation trends. The system can also only effectively identify misinformation pertaining to a single health topic at a time, as determined by the keywords defined by the users in the query for data extraction. As a result, the system currently only pulls the publications that directly contain the keywords of the health topic. In this iteration, it cannot identify the publications that retain context when read within a conversation stream but do not contain the actual keywords of the health topic. It is also important to acknowledge that other social media giants such as Facebook and YouTube are major sources of digital misinformation. Future work will also focus on integrating data from multiple social media giants through custom data extraction frameworks into the dashboard for multiplatform analysis. Such multiplatform analysis will involve separate dashboards for each platform and a single dashboard for all the platforms combined.

### Conclusions

The novel U-MAS pipeline has the potential to collect, detect, and analyze the rapidly increasing amounts of misinformation proliferated on social media related to a particular topic or set of related topics. Once an ecosystem of expert systems is in place, analytics dashboards and anomaly or risk detection alerts may be used to determine when specific topics are experiencing considerable trends of health misinformation, assisting in identifying and mitigating falsehoods through prompt governmental intervention.

## Appendix

Multimedia Appendix 1 Real-time, interactive analytics front end of the system using Kibana.

## Abbreviations

API: application programming interface
BERT: Bidirectional Encoder Representations from Transformers
BLOB: Binary Large Objects
LDA: latent Dirichlet allocation
MCC: Matthews correlation coefficient
NDJSON: Newline Delimited JSON
NLP: natural language processing
ReLu: rectified linear unit
U-MAS: UbiLab Misinformation Analysis System
VADER: Valence Aware Dictionary and Sentiment Reasoner
VM: virtual machine

## Data Availability

The raw data used for fluoride misinformation use case have been anonymously disclosed in an open data repository [68]. The raw data for the other use cases may be available upon written request.
