# Supplementary material for: Tweeting for Health Using Real-time Mining and Artificial Intelligence–Based Analytics: Design and Development of a Big Data Ecosystem for Detecting and Analyzing Misinformation on Twitter
Source: J Med Internet Res. 2023 Jun 9;25:e44356. doi: 10.2196/44356 (PMC10337356; doi:10.2196/44356)
Supplement: Multimedia Appendix 1 [file jmir_v25i1e44356_app1.docx]

**Tweeting for Health using Real-Time Mining and AI-Based Analytics: Design & Development of as Misinformation Data Ecosystem for Twitter**

Plinio P Morita^1,4,5,6,7#^, Irfhana Zakir Hussain^1,2 #^, Jasleen Kaur^1#^, Matheus Lotto^1,3^, Zahid Ahmad Butt^1^

^1^School of Public Health Sciences, Faculty of Health, University of Waterloo, Waterloo, ON, Canada

^2^Department of Data Science and Business Systems, School of Computing, College of Engineering and Technology, SRM Institute of Science and Technology, Kattankulathur, India

^3^Department of Pediatric Dentistry, Orthodontics and Public Health, Bauru School of Dentistry, University of São Paulo, Bauru, Brazil

^4^Research Institute for Aging, University of Waterloo, Waterloo, ON, Canada

^5^Institute of Health Policy, Management, and Evaluation, University of Toronto, Toronto, ON, Canada

^6^Department of Systems Design Engineering, University of Waterloo, Waterloo, ON, Canada

^7^eHealth Innovation, Techna Institute, University Health Network, Toronto, ON, Canada

**^#^ These authors contributed equally to this work and share the first authorship**

**Corresponding Author:**

Plinio P Morita, Peng, MSc, PhD

School of Public Health Sciences, Faculty of Health, University of Waterloo

200 University Avenue West, Waterloo, ON, N2L 3G1, Canada

Phone: 1 5198884567 ext 41372

Email address: [plinio.morita@uwaterloo.ca](mailto:plinio.morita@uwaterloo.ca)

**SUPPLEMENTARY FIGURES**

**Figure 1(a-e)***: Real-Time, Interactive Analytics Front-End of the System using Kibana.

**
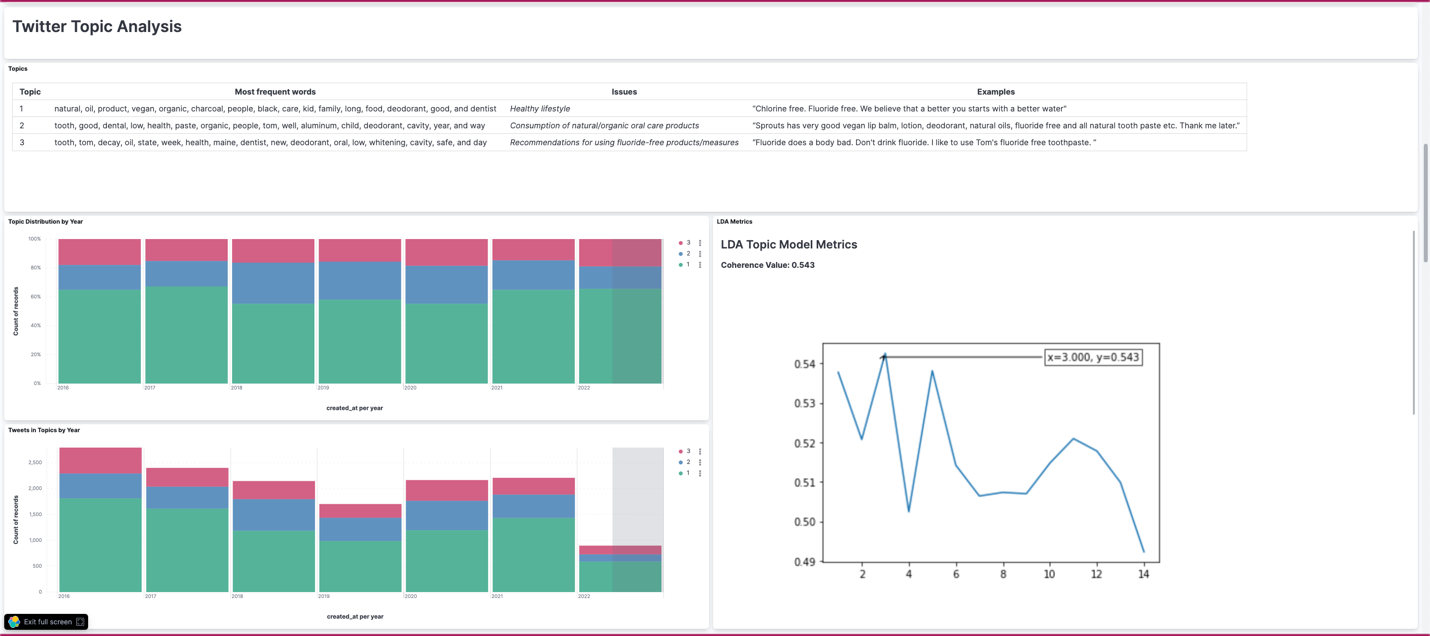
**

**(a)**

**
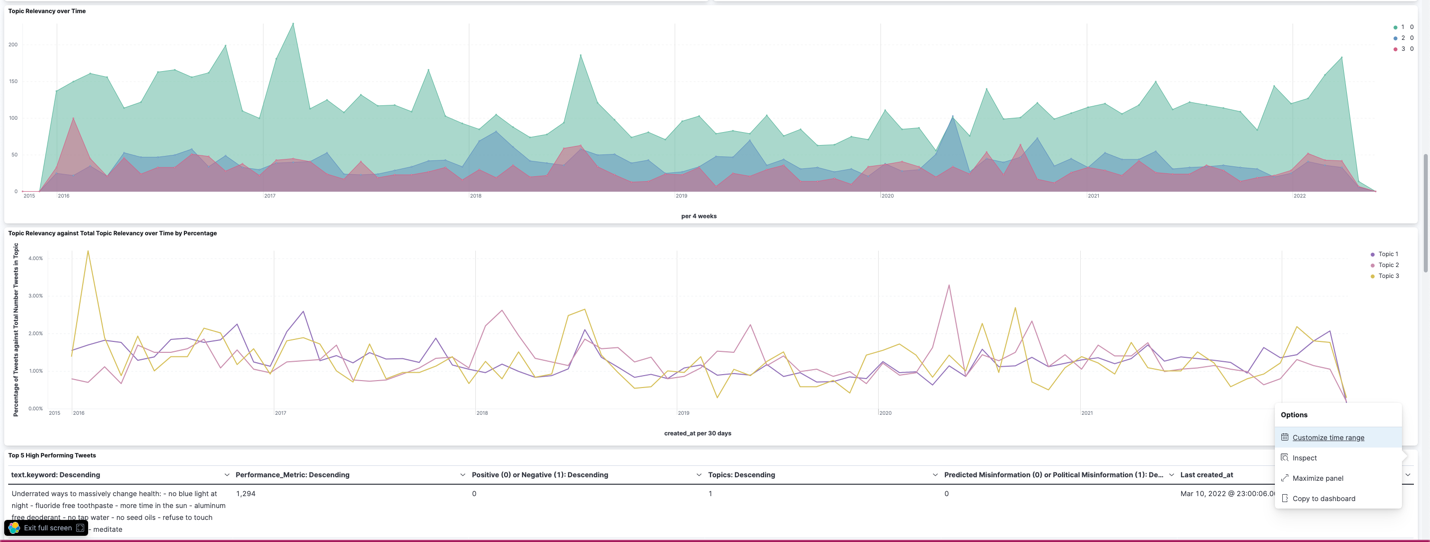
**

**(b)**

**
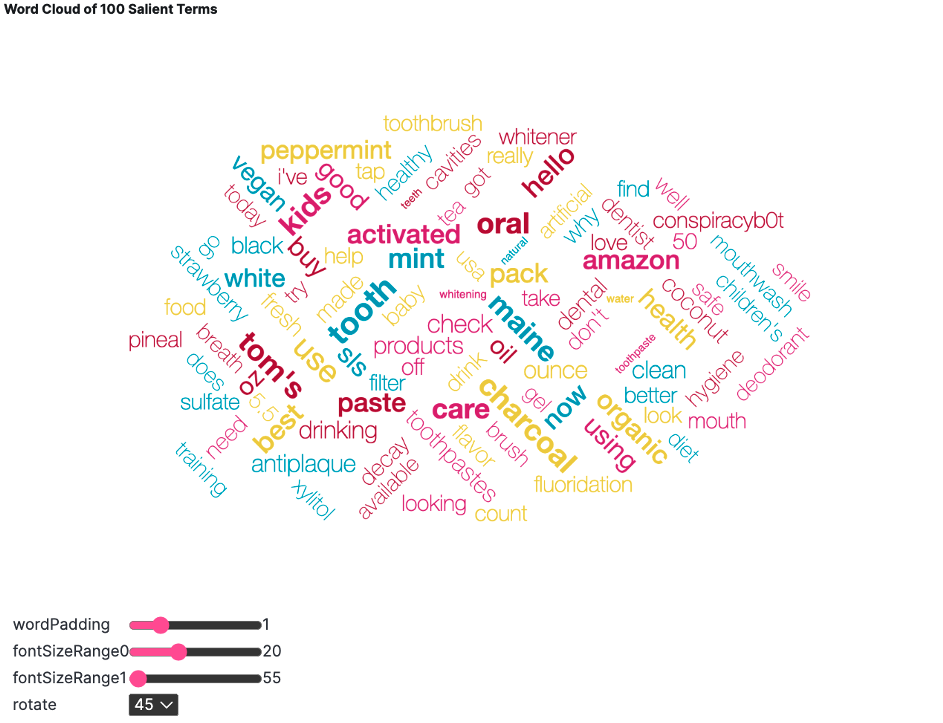
**

**(c)**

**
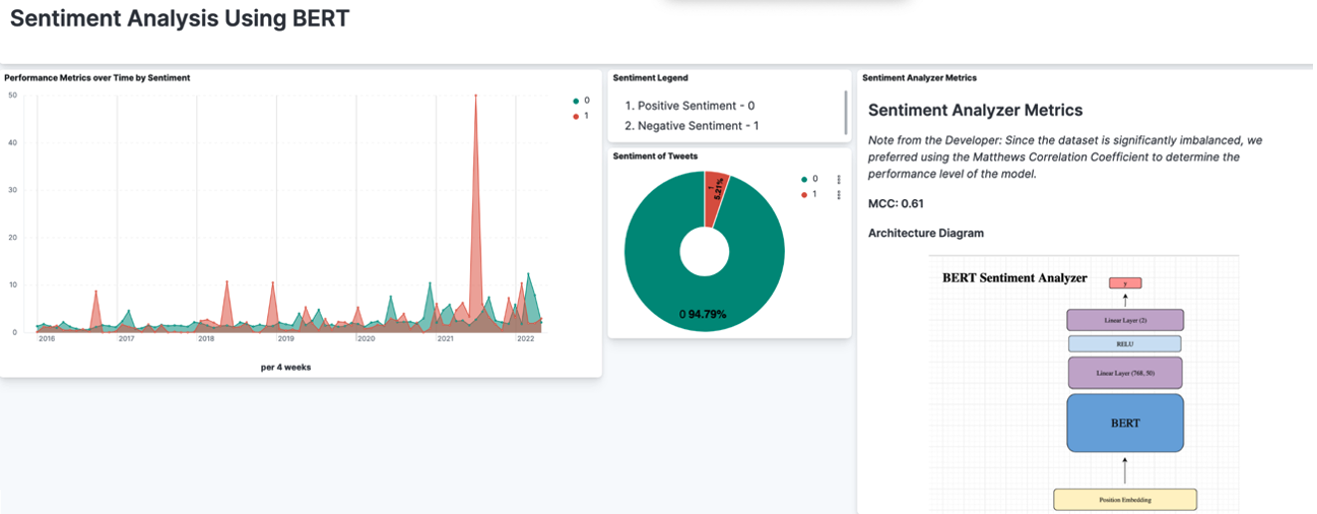
**

**(d)**

**
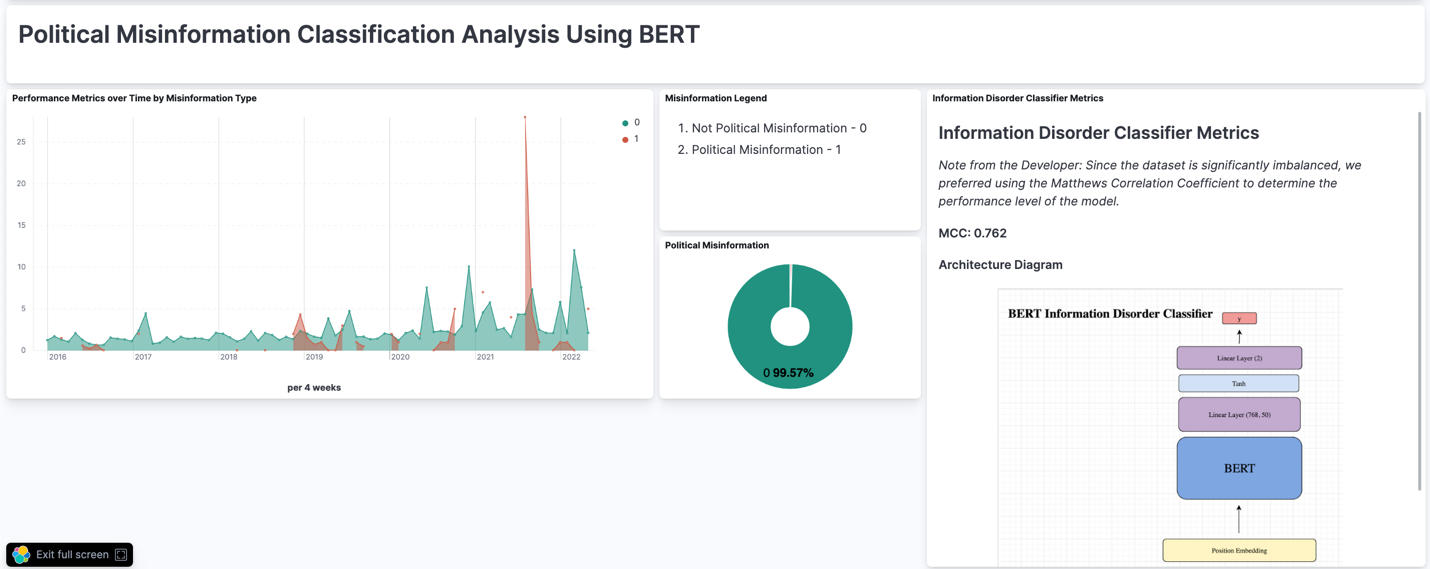
**

**(e)**
